# The relationship between preoperative anxiety and postoperative recovery index in laparoscopic abdominal surgery patients

**DOI:** 10.1590/1806-9282.20252154

**Published:** 2026-06-29

**Authors:** Havva Bozdemir, Nihat Çiftci, Züleyha Şimşek Yaban, Yasemin Özhanli, Abdullah Güneş

**Affiliations:** 1 Kocaeli University, Faculty of Health Sciences – Kocaeli, Turkey.; 2 Kocaeli University, Institute of Health Sciences – Kocaeli, Turkey.; 3 Kocaeli City Hospital, Department of Register Nursing – Kocaeli, Turkey.; 4 Kocaeli City Hospital, General Surgery Clinic – Kocaeli, Turkey.

**Keywords:** Anxiety, Postsurgical recoveries, Surgical procedures, Acute pain, General surgery

## Abstract

**OBJECTIVE:**

The aim of this study was to determine the relationship between preoperative anxiety and postoperative pain and recovery index in patients undergoing laparoscopic abdominal surgery.

**METHODS:**

This study was descriptive and correlational. The study population consisted of 115 patients who underwent laparoscopic abdominal surgery in the General Surgery Department of a state hospital in Kocaeli. Permission to conduct the study was obtained from the institutions where the study was to be conducted, and ethical approval was obtained from the Scientific Research Ethics Committee of Kocaeli City Hospital. Data were collected using the Visual Analogue Scale pain scale, the Amsterdam Preoperative Anxiety and Information Score, the State–Trait Anxiety Inventory, and the Postoperative Recovery Index.

**RESULTS:**

A moderate, positive, and statistically significant correlation was found between State–Trait Anxiety Inventory-State Anxiety scores and Postoperative Recovery Index total scores. Furthermore, State–Trait Anxiety Inventory-State Anxiety scores were found to be significantly correlated with the subscales of the Postoperative Recovery Index. The correlations with these subscales were as follows: Physical Activities, Bowel Symptoms, General Symptoms, Desire/Urge, and Psychological Symptoms.

**CONCLUSION:**

It was concluded that as the preoperative anxiety level of patients undergoing laparoscopic abdominal surgery increased, they experienced difficulties in postoperative recovery and as the patients’ well-being decreased.

## INTRODUCTION

The care of surgical patients involves a more challenging and prolonged postoperative period compared to other stages of the healthcare process^1^. Therefore, recovery depends not only on the surgeon but also on the combined efforts of the various healthcare professionals involved in the patient’s treatment. All professions offer specialized knowledge and a unique perspective to help meet the complex needs of individuals during the postoperative rehabilitation process^2^.

Monitoring the patient’s condition, preventing complications, providing emotional support, and effectively coordinating care are the fundamental objectives of nursing care in postoperative care. Nurses are often the healthcare providers with whom patients communicate most during recovery, and therefore, their involvement in the treatment process is key to a smooth recovery^3^. Studies evaluating pre- and postoperative anxiety have identified the presence of preoperative anxiety^4,5,6,7^. It has been reported that recovery varies between different days and different types of surgery^8,9,10^. Regardless of the type of surgical intervention, the decision to undergo surgery causes anxiety in individuals. Anxiety and fear can prolong recovery because they exacerbate anxiety-related complications in the postoperative period^5,11^.

Although there are studies in Turkey addressing anxiety and postoperative recovery in surgical patients, data specific to patients undergoing laparoscopic abdominal surgery are insufficient. Despite the fact that the pain and recovery dynamics of laparoscopic surgery differ from open surgery, the relationship between preoperative anxiety and postoperative pain and recovery in this patient group has not been systematically investigated. Therefore, to fill this literature gap and provide evidence-based contributions to clinical practice, this study was planned to evaluate the relationship between preoperative anxiety on postoperative pain and recovery index in patients undergoing laparoscopic abdominal surgery.

## METHODS

The study was descriptive and cross-sectional. The study population consisted of patients who underwent laparoscopic abdominal surgery at the general surgery department of Kocaeli City Hospital between June 26, 2025 and August 11, 2025. The sample size was determined using the bivariate correlation test in the G Power 3.1.9.4. programme. The minimum sample size was determined to be 115 patients for a 0.05 error margin, 0.95 power, and 0.3 (medium) effect size for the relationship between variables.

Patients who underwent laparoscopic abdominal surgery, volunteered for the study, were over 18 years of age, had no psychiatric problems, could communicate in Turkish, and had no surgical experience were included in the study.

Data collection tools for the study included Visual Analogue Scale (VAS), the Amsterdam Preoperative Anxiety and Information Score (APAIS), the State–Trait Anxiety Inventory (STAI), and the Postoperative Recovery Index (PoRI).

Descriptive Characteristics Form: This consists of questions regarding age, gender, medical diagnosis, and type of surgery performed.

VAS: The VAS is an extremely simple, effective, reproducible, and minimally instrument-dependent measurement tool. The scale consists of a 10-cm-long line drawn vertically or horizontally. The patient is asked to mark the point on this line that corresponds to the intensity of their pain.

State Anxiety Inventory (STAI): For the state anxiety level (STAI) used for anxiety states with clinically significant symptoms, 0–40 is considered normal and above 41 is considered anxious. This is because the STAI shows how the person feels regardless of their current situation and circumstances. The scores obtained from the STAI theoretically range from 20 to 80 points. As the average score increases, state anxiety increases^12^. For this study, the Cronbach’s alpha value was found to be 0.701.

The APAIS: It is one of the tests used to assess preoperative anxiety. In this test, the source of anxiety is divided into three categories: anxiety about surgery, anxiety about anesthesia, or anxiety caused by a lack of information. To assess anxiety, it consists of six items and numerical values based on a 5-point Likert scale. The lowest score is 6, and the highest score is 30^13^. For this study, the Cronbach’s alpha value was found to be 0.901.

PoRI: The PoRI consists of 25 items. The PoRI has five subscales: psychological symptoms, physical activities, general symptoms, bowel symptoms, and desire-motivation symptoms. High scores on the index reflect greater difficulty in postoperative recovery, while low scores indicate easier postoperative recovery^8^. The Cronbach’s alpha value for this study was found to be 0.932.

### Data analysis

Descriptive statistics (percentage, frequency, mean, and standard deviation) were used to examine the personal and professional characteristics of the research data and the scale scores. The internal consistency coefficient was calculated to assess the reliability of the scales. When examining the distribution of variables using the Shapiro-Wilk test, histogram graph, and skewness and kurtosis measurements (+1.5, −1.5), the distribution of the data was found to be non-homogeneous. Independent sample t tests, Kruskal-Wallis H test, and Pearson correlation tests were used in the analysis of the data. A p<0.05 was considered statistically significant. Multiple linear regression analysis was performed to determine the predictors of the PoRI. The analysis was conducted in two stages. Model 1 included age and gender variables. Model 2 added scores from the State–Trait Anxiety Inventory (STAI) and the APAIS, which assess preoperative anxiety levels. Results were reported as odds ratios (OR) with 95%CI and were considered statistically significant at p<0.05.

### Findings

The mean age of the participants was 50.43±4.14, 57.4% were female, and all were married. 63.5% of patients underwent cholecystectomy, 21.7% inguinal hernia repair, 9.6% umbilical hernia repair, 2.6% hiatal hernia repair, and 2.6% total colectomy.

According to the findings obtained in this study, the mean preoperative STAI-State Anxiety total score of the participants was 24.17±4.91. The APAIS total score was determined to be 13.81±6.29. It was determined that the patients’ preoperative anxiety was at a moderate level. The PoRI total score was 40.79±11.57 (Min: 25, Max: 69). When evaluated based on their total scores during the postoperative recovery process, it was determined that participants experienced little difficulty but had significant difficulty with physical activities.

A moderate, positive, and statistically significant correlation was found between STAI-State Anxiety scores and the total score of the PoRI (r=0.687, p<0.01). Furthermore, STAI-State Anxiety scores were also found to be significantly correlated with the subscales of the PoRI. The correlation coefficients with these subscales are as follows: Physical Activities (r=0.651), Bowel Symptoms (r=0.396), General Symptoms (r=0.621), Craving/Desire (r=0.565), and Psychological Symptoms (r=0.657). The findings indicate that as state anxiety levels increase, postoperative recovery is negatively affected.

A moderate, positive, and statistically significant correlation was found between the APAIS total score and the PoRI total score (r=0.589, p<0.01). When examining the correlation between APAIS subscales and PoRI, a weak correlation was found between anesthesia anxiety and PoRI (r=0.470), moderate (r=0.615) positive and significant relationship between surgical anxiety and PoRI, and again a moderate (r=0.527) positive and significant relationship between the desire for information and PoRI. In particular, high levels of surgical anxiety show stronger relationships with various dimensions of the recovery process (Table 1).

**Table 1:** Relationship between Preoperative State Anxiety Scale Score, Amsterdam Preoperative Anxiety and Information Scale, and Postoperative Recovery Index.

Variables^¥^	ASII total	ASII subscales				
		Physical activities	Bowel symptoms	General symptoms	Desire-craving	Psychological symptoms
STAI-State Anxiety	0.687^**^	0.651^**^	0.396^**^	0.621^**^	0.565^**^	0.657^**^
APAIS total	0.589^**^	0.586^**^	0.486^**^	0.399^**^	0.438^**^	0.505^**^
APAIS-Anesthesia Anxiety	0.470^**^	0.455^**^	0.451^**^	0.428^**^	0.425^**^	0.394^**^
APAIS-Surgical Anxiety	0.615^**^	0.598^**^	0.463^**^	0.429^**^	0.392^**^	0.592^**^
APAIS-desire to acquire information related to anesthesia and surgery	0.527^**^	0.532^**^	0.475^**^	0.282	0.423^**^	0.386^**^

STAI: State–Trait Anxiety Inventory; APAIS: Amsterdam Preoperative Anxiety and Information Scale; PoRI-TR: Postoperative Recovery Index

^¥^Pearson correlations

^**^p<0.01.

A negative correlation was found between participants’ age and preoperative APAIS (r=−0.308, p=0.001) and STAI-I (r=−0.166, p=0.076) scores, with only the correlation with APAIS being statistically significant. No significant relationship was observed between age and postoperative PoRI (r=0.031, p=0.740) and VAS (r=−0.024, p=0.802) scores.

In the comparison based on the gender variable, women had significantly higher preoperative APAIS (t=4.722, p=0.005), STAI-I (t=5.046, p<0.001), postoperative PoRI (t=2.356, p=0.020), and VAS (t=2.519, p=0.013) scores than men.

According to the results of the variance analysis conducted for the surgery type variable, there were significant differences between the groups in preoperative APAIS (KW=31.58, p=0.000), STAI-I (KW=27.29, p=0.000), and PoRI (KW=12.75, p=0.013) scores. However, the difference in VAS scores was at the threshold of significance and was not found to be statistically significant (KW=2.148, p=0.080). According to post-hoc analyses, the “Inguinal hernia” and “Colorectal surgery” groups had significantly lower APAIS and STAI-I scores, while the “Umbilical hernia” group had the highest anxiety levels. Similarly, postoperative PoRI scores were also found to be lower in the “Inguinal hernia” group (Table 2).

**Table 2:** Comparison of Preoperative State-Trait Anxiety Inventory-State Anxiety, Amsterdam Preoperative Anxiety and Information Score, Postoperative Recovery Index, and Visual Analogue Scale-Pain Scores according to patient characteristics.

Variables		n (%)	Preop-APAIS	Preop-STAI-I	Postoperative PoRI-TR	Postoperative VAS
Age^β^		115 (100)				
	r		-0.30	-0.16	0.03	-0.02
	p-value		0.001^*^	0.07	0.74	0.80
Gender	Female	66 (57.3)	16.00±6.60	25.86±5.40	42.93±12.10	3.45±1.64
	Male	49 (42.7)	10.85±4.40	21.89±2.93	37.89±10.22	2.71±1.42
	t^*^		4.720	5.046	2,356	2,519
	p-value		0.005	0.000	0.020	0.013
Type of surgery^¥^	Cholecystectomy^a^	73 (63.5)	14.94±6.55	25.15±5.55	43.02±12.23	3.35±1.68
	Inguinal hernia^b^	25 (21.7)	8.84±2.95	21.04±1.42	34.72±9.22	2.44±1.29
	Umbilical hernia^c^	11 (9.6)	18.36±4.12	25.54±2.97	40.36±10.25	3.63±1.36
	Hiatal hernia^d^	3 (2.6)	15.66±2.30	25.66±0.57	38.66±5.77	2.66±1.15
	Total colectomy^e^	3 (2.6)	9.00±1.73	20.00±0.33	40.66±4.04	2.33±1.15
	χ KW		31.58	27.29	12.75	2,148
	p-value		0.000	0.000	0.013	0.080
			b=e<a=d<c	b<d=c=e<a	b<d=c=e<a	

STAI: State–Trait Anxiety Inventory; APAIS: Amsterdam Preoperative Anxiety and Information Scale; PoRI-TR: Postoperative Recovery Index; VAS: Visual Analogue Scale

^*^independent sample t-test

^¥^Kruskal-Wallis H Test

^β^Pearson correlations. The analysis was marked with the letters a, b, c, and d to allow for interpretation.

The results of the multiple linear regression analysis examining the determinants of the PoRI are presented in Table 3. In the first model (Model 1), age and sex were entered as predictors. Model 1 was statistically significant (R=0.230, R^2^=0.053, F=3.136, p=0.047), explaining 5.3% of the variance in postoperative recovery outcomes. Age was not significantly associated with PoRI scores in this model (B=0.065, β=0.081, p=0.393). In contrast, female sex demonstrated a significant positive association with PoRI scores (B=5.439, β=0.233, p=0.013), indicating poorer postoperative recovery among female patients.

**Table 3:** Moderation effect results.

Variable	Posrop-PoRI					
	Model 1			Model 2		
	B(β)	p-value	95%CI	B(β)	p-value	95%CI
Constant	45.263	0.000	36.291 (54.235)	5.596	0.357	-17.575 (6.383)
Age (years)	0.065 (0.081)	0.393	-0.085 (0.217)	0.170 (0.211)	0.002	0.061 (0.280)
Sex (female)	5,439 (0.233)	0.013	-9.782 (1.096)	1.812 (1.658)	0.277	-1.475 (5.098)
Preop-STAI				1.036	0.000	0.547 (1.524)
Preop-APAIS				0.736	0.000	0.343 (1.130)
	R=0.230 R^2^=0.053 F _(3.136)_ =p=0.047			R=0.742 R^2^=0.551 F _(33.761)_ =p=0.000 Durbin-Watson: 1.643		

Model 1: age, sex, Posop-PoRI, Model 2: Model 1 variable plus Preop-STAI, Preop-APAIS, B unstandardized regression coefficient, β standardized regression coefficient, Adj. R2 adjusted R-squared, CI: confidence interval; PoRI: Postoperative Recovery Index; STAI: State–Trait Anxiety Inventory; APAIS: Amsterdam Preoperative Anxiety and Information Scale.

In Model 2, preoperative anxiety measures—preoperative State–Trait Anxiety Inventory (STAI) and preoperative APAIS scores—were added to the model. This extended model showed a marked improvement in explanatory power and was highly statistically significant (R=0.742, R^2^=0.551, F=33.761, p<0.001), accounting for 55.1% of the variance in PoRI scores.

Within Model 2, age emerged as a significant and positive predictor of postoperative recovery impairment (B=0.170, β=0.211, p=0.002; 95%CI 0.061–0.280), whereas the relationship of sex was attenuated and no longer statistically significant (B=1.812, p=0.277). Both preoperative anxiety variables demonstrated strong and independent associations with PoRI scores. Higher preoperative STAI scores were significantly associated with increased postoperative recovery difficulty (B=1.036, p<0.001; 95%CI 0.547–1.524), as were higher preoperative APAIS scores (B=0.736, p<0.001; 95%CI 0.343–1.130). The Durbin–Watson statistic (1.643) suggested no evidence of autocorrelation among residuals, confirming that the assumption of independence was satisfied. These findings indicate that increasing age and elevated levels of preoperative anxiety are associated with greater postoperative recovery impairment. The inclusion of anxiety-related variables substantially enhanced the explanatory capacity of the model, underscoring the pivotal role of preoperative psychological status in postoperative recovery.

## DISCUSSION

Surgery is a situation that can cause anxiety and can lead to an increase in perioperative complications, prolonged hospital stay, and delayed recovery^6,7,14^. This study presented the relationship between preoperative anxiety and postoperative pain and recovery index in patients undergoing laparoscopic abdominal surgery.

In the study, when the preoperative anxiety levels of patients undergoing laparoscopic abdominal surgery were assessed using STAI-I and APAİS, they were found to be moderate. Anxiety related to surgery was found to be high. The results of studies conducted with patients undergoing different types of surgery in the literature are consistent with our findings^5,10^. In studies, it was found that patients experienced mild anxiety before surgery^15,16^. A meta-analysis reported a global prevalence of preoperative anxiety of 48%^17^. The assessment of preoperative anxiety is of great importance for the perioperative process. Assessing anxiety is very important for patients undergoing surgical intervention, and early diagnosis can positively affect the treatment process. A meta-analysis indicated that preoperative counseling and comprehensive education programmes are effective in reducing anxiety in patients during the postoperative period^18^. Postoperative bleeding and fear of surgical complications can cause anxiety in patients^5,14^.

When participants were assessed based on their total scores during the postoperative recovery period, it was determined that they experienced significant difficulties in physical activities. A meta-analysis study found that patients undergoing orthopedic surgery experienced preoperative anxiety, which negatively affected the recovery process and patient satisfaction, and that social support was very important in reducing anxiety^19^. Our study findings indicate that as state anxiety levels and anxiety increase, postoperative recovery is negatively affected. This finding is consistent with the findings of a study conducted on patients undergoing cardiovascular surgery. However, the same study noted that cardiac surgery patients had high anxiety levels and experienced difficulties in recovery^20^. This difference is explained by the type of surgery and the collection of data during the COVID-19 pandemic. In addition, various factors such as surgical technique, duration, and type of intervention affect recovery^21^. However, despite different surgical types, studies reporting that recovery after surgery decreases as anxiety levels increase are consistent with our research findings^22,23^. Although laparoscopic surgery has fewer postoperative complications, it has not been shown to affect preoperative anxiety levels.

Our study found that female patients were more anxious and experienced greater difficulty in recovery. However, another study reported that gender did not affect the recovery index^24^. The higher lifetime prevalence of anxiety disorders among women may be associated with their greater tendency to express health-related concerns, their increased sensitivity to uncertainty and loss of control, and their higher perception of threat regarding surgical pain, risk of complications, and fear of anesthesia.

Another finding from our research is that age did not affect postoperative recovery. Contrary to our findings, it has been reported that elderly patients experience more difficulty in postoperative recovery^24^. We believe that these differences may stem from differences in the type of surgery between the sample groups. The fact that the mean age did not create a significant difference in the postoperative recovery process in our study can be explained by the mean age being 50 and the middle age period being a time when physiological reserves are largely preserved, and the burden of comorbidity is more limited compared to older age groups. We believe that the general health status, comorbidities, and functional capacity of individuals are more decisive factors in recovery than chronological age.

In the management of anxiety during the preoperative preparation phase, providing individualized care is essential for developing nurse-led care packages. Therefore, individualized care planning should be evidence-based and supported by research findings.

## CONCLUSION

The findings of this study demonstrate that preoperative anxiety has a significant and clinically meaningful relationship with postoperative recovery in patients undergoing laparoscopic abdominal surgery. High levels of surgery-specific anxiety, in particular, negatively impact the physical, emotional, and functional dimensions of recovery. Therefore, routine assessment of patients’ anxiety levels in the preoperative period using valid and reliable scales such as STAI-I and APAIS should be considered an important component of individualized care. The higher anxiety levels and more challenging recovery process in female patients highlight the need for targeted psychosocial support and counseling interventions for this patient group. In line with these findings, it is recommended that structured education, information, and support programs aimed at reducing preoperative anxiety be integrated into clinical practice. The most important strength of this study is that the assessment of anxiety using both general (STAI-I) and surgery-specific (APAIS) scales allowed for a multidimensional examination of anxiety and increased the clinical validity of the findings. Furthermore, the fact that postoperative recovery is assessed holistically through a recovery index, rather than solely focusing on pain, makes the study’s results more valuable in terms of clinical decision-making processes.

Some limitations of this study include the following: first, its observational and cross-sectional nature does not allow for establishing a causal relationship between preoperative anxiety and postoperative recovery; second, the assessment of anxiety levels using self-report scales introduces the possibility of bias stemming from individual perceptions and response tendencies; and third, the fact that the study was conducted at a single center may limit the generalizability of the findings.

## Data Availability

The datasets generated and/or analyzed during the current study are available from the corresponding author upon reasonable request.
